# Household knowledge and practices concerning malaria and indoor residual spraying in an endemic area earmarked for malaria elimination in Iran

**DOI:** 10.1186/s13071-017-2548-z

**Published:** 2017-12-06

**Authors:** Abdoulhossain Madani, Moussa Soleimani-Ahmadi, Sayed Hossein Davoodi, Alireza Sanei-Dehkordi, Seyed Aghil Jaberhashemi, Mehdi Zare, Teamur Aghamolaei

**Affiliations:** 1 Social Determinants in Health Promotion Research Center, Hormozgan University of Medical Sciences, Bandar Abbas, Iran; 2 Department of Medical Entomology and Vector Control, Faculty of Health, Hormozgan University of Medical Sciences, P.O. Box: 79145-3838, Bandar Abbas, Iran; 3 Department of Nutrition Research, National Nutrition and Food Technology Research Institute, Faculty of Nutrition Sciences and Food Technology, Shahid Beheshti University of Medical Sciences, Tehran, Iran; 4 Bashagard Health Center, Hormozgan University of Medical Sciences, Bashagard, Iran; 5 Department of Occupational Health Engineering, Faculty of Health, Hormozgan University of Medical Sciences, Bandar Abbas, Iran

**Keywords:** Malaria, Knowledge, Practices, Household, Indoor residual spraying, Iran

## Abstract

**Background:**

Indoor residual spraying of insecticide (IRS) is a key intervention for reducing the burden of malaria infection. Effectiveness and success of this strategy are to a considerable extent dependent on knowledge and practice of the target community regarding the IRS. Iran has entered the malaria elimination phase, and IRS has been considered as the main strategy for malaria vector control. Therefore, this study was conducted to determine the household knowledge and practices about malaria and IRS in Bashagard County, one of the malaria-endemic areas in the southeast of Iran.

**Methods:**

A community-based cross-sectional survey was conducted among 420 households in Bashagard County. The participants who were selected using a two-stage randomized cluster sampling procedure were subjected to a tested structured questionnaire. During the survey, direct observations were made concerning the use of IRS as well as housing conditions. The data were coded and analysed using SPSS version 19.

**Results:**

Knowledge levels about malaria as a disease and the mosquito as its vector were high and of equal magnitude (85.5% and 85.4%, respectively), while knowledge levels of IRS were even higher (91.6%). The main source of households’ information about malaria and IRS was primarily community health workers (73.3%). Despite positive perceptions towards IRS only 26.7% of respondents had sprayed their houses which is lower than the WHO targeted coverage of 80%. Respiratory disorders and headache (33.3%), food contamination (24.9%), discolouring of inner house walls (17.7%), difficulty in furniture’s movement (13.8%), and unpleasant odour (10.4%) were the main reasons for IRS refusal.

**Conclusion:**

There is a discrepancy between knowledge about symptoms and the transmission route of malaria and control practices related to IRS use. Therefore, IRS campaigns accompanied with education for behaviour change should be considered to ensure householders’ participation and cooperation in the IRS programme. Moreover, continuous evaluation and monitoring of IRS as well as conducting more surveys on knowledge, attitude, and practices are recommended to improve malaria control measures and to identify indicators for effective, successful, and sustainable malaria elimination programme.

## Background

Malaria remains a major contributor to worldwide disease burden and is currently endemic in 91 countries [1]. In 2015, approximately 212 million new cases of malaria were diagnosed, and about 429,000 people died from malaria worldwide [1].

In Iran, the malaria eradication campaign was initiated in 1951 and changed to malaria control in 1985 as a result of constraints and challenges [2]. Iran has been in the current elimination phase since 2010. In 2009, the number of malaria cases in Iran were 6122, and it was reduced to 720 in 2015 [1, 3].

Iran, as in other malaria-endemic countries, has employed effective vector control measures including indoor residual spraying (IRS), long-lasting insecticidal nets (LLINs), and application of larvicides as the main vector control interventions [4–6]. Challenges to the elimination of malaria in low socioeconomic areas, such as Bashagard County, include the inability to sustain control programmes due to community perceptions and practice for malaria control. Promotion of community participation through health education and communication programmes can be considered as a strategy against these challenges.

IRS is the large-scale application of insecticides to spray the interior of homes for killing mosquitoes. It remains one of the main components of the malaria control strategy, which aims to prevent parasite transmission through interventions targeting Anopheline vectors [7, 8]. IRS has been employed to eliminate malaria from different malaria endemic areas including Europe, Asia, Latin America, and Africa [9]. National malaria control programmes in the 91 endemic countries reported that 106 million people worldwide were protected by IRS in 2015 [1]. Several factors which influence the effectiveness of IRS interventions include spraying coverage, type of insecticide, type and situation of houses, community awareness and cooperation, household acceptance, and informing the households about the programme benefits and spraying time during the spraying campaign [10]. Spraying coverage is also dependent on the household’s perception of the effectiveness of IRS programme against mosquitoes and other nuisance insects, as well as the number and intensity of unwanted side effects [8]. The perceived side effects of indoor insecticide spraying can decrease the acceptance of these types of interventions [11]. It is thus necessary to understand community knowledge about house spraying for the IRS programmes to be successful [12].

Previous studies have demonstrated that communities have positive expectations when interventions such as IRS are introduced [8, 11]. However, they may refuse IRS due to their concerns about IRS with regards to chemicals being a health hazard or doubts about its effectiveness [11, 13].

According to the recent reports, almost all areas of Iran are considered as malaria-free, except some regions in the south-east of the country which are still considered as endemic malaria area [1, 14]. Bashagard is one of the malaria-endemic regions in this area with two seasonal peaks in autumn and spring. In this county 1406 cases of malaria were reported during 2008–2015, out of which 1394 (99.15%) and 12 (0.85%) cases were attributed to *Plasmodium vivax* and *P. falciparum*, respectively (Bashagard Health Center, unpublished data, 2015). Six species including *Anopheles stephensi*, *An. fluviatilis*, *An. dthali*, *An. culicifacies*, *An. superpictus* and *An. pulcherrimus* have been reported as malaria vectors in this County [15, 16]. One of the WHO recommended strategic approaches for malaria elimination by the use of indoor residual spraying programmes which are highly dependent on pyrethroid insecticides [4]. Regarding this recommendation, IRS has been focused on, by Iranian Ministry of Health and Medical Education as the main component of the malaria vector control intervention strategy [7]. The target of National Malaria Control Programme is to cover at least 85% of all targeted households to achieve community coverage sufficient to interrupt transmission of malaria [1].

Community participation is the key component of malaria elimination programmes, and improved community knowledge of malaria control methods can promote preventive practices against malaria [14, 17]. To achieve efficient IRS coverage, a key factor is identifying and addressing the behavioural factors that may lead to IRS refusal. Increasing the community knowledge about malaria and IRS will lead to behavioural changes which would help in designing sustainable malaria control programmes [13]. In line with assuring for sustainability of the malaria elimination programme in Iran, this study was conducted to determine the community knowledge and practice about malaria and IRS, as a preventive strategy, in Bashagard County, southeast of Iran.

## Methods

### Study area

**Fig. 1:**
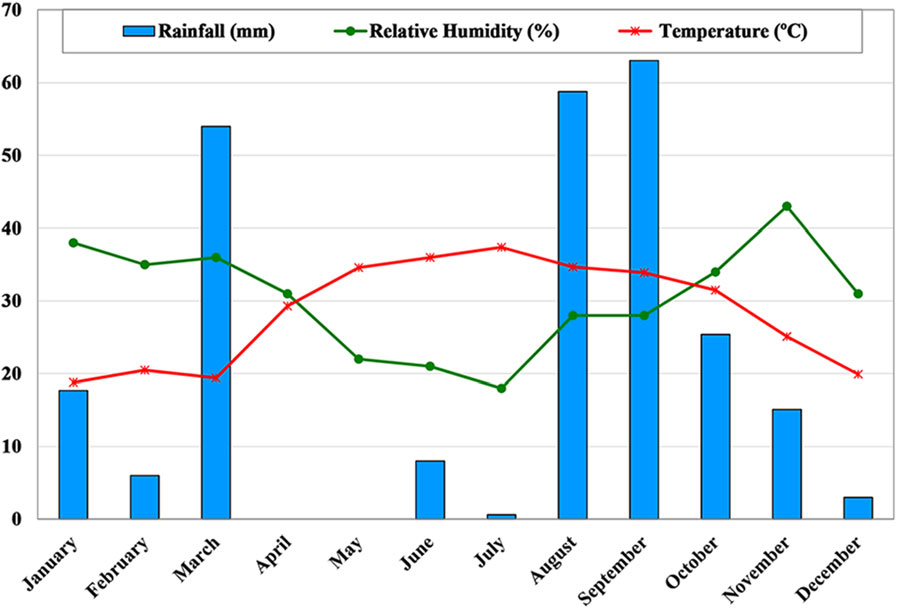
Average of meteorological parameters during 2015–2016 in Bashagard County, southeastern Iran

**Fig. 2:**
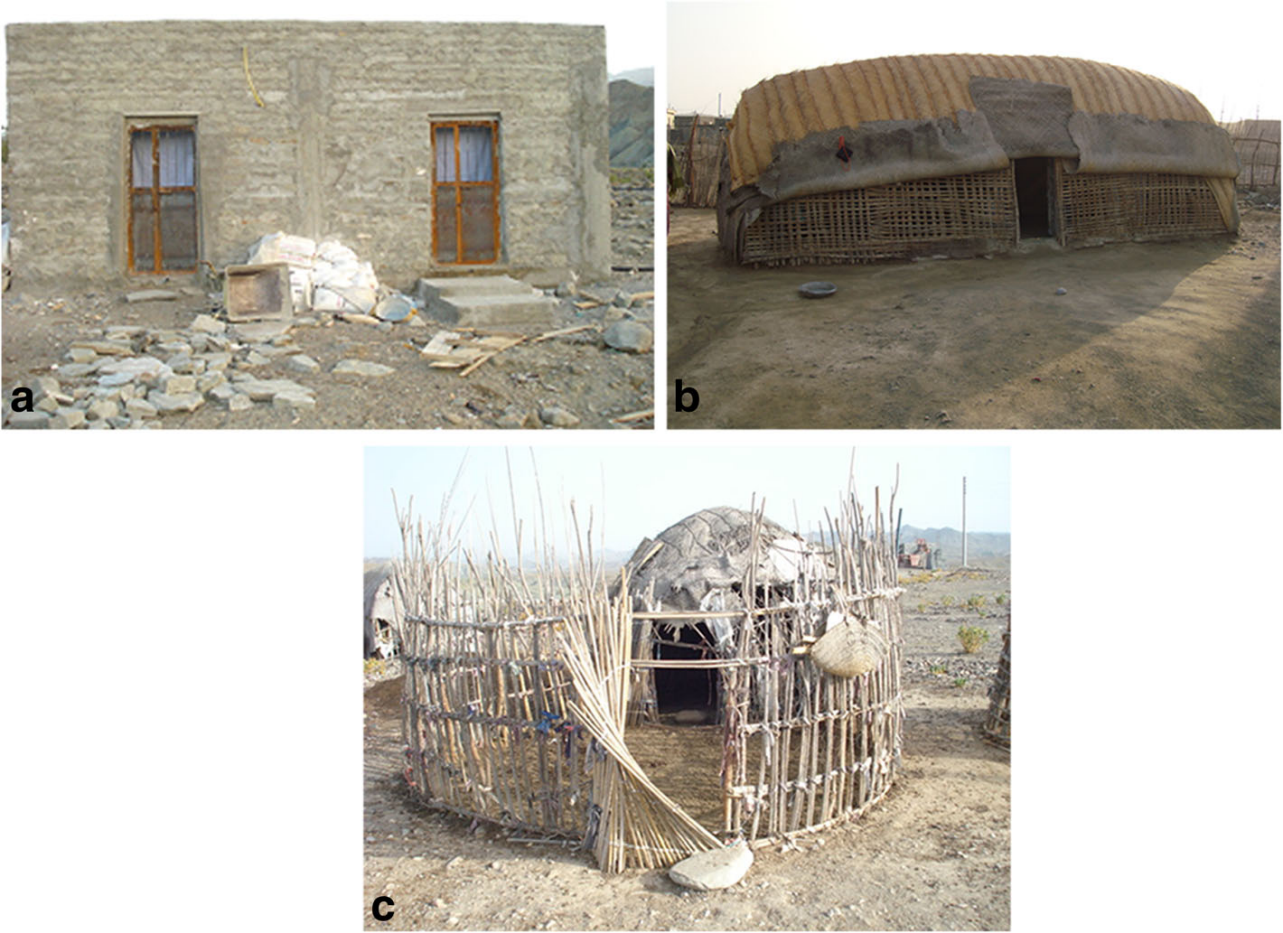
Typical habitats of human and animal in Bashagard County, southeastern Iran. Cemented brick houses (**a**), sheds made with palm leaves (**b**) and domestic animal shed (**c**)

Bashagard County is located between 26°04′–26°58′N latitudes and 57°23′–59°02′E longitudes in Hormozgan province, southeastern Iran, with an estimated population of 40,037 individuals in 2015. The climate in Bashagard is categorized as tropical dry, with mean annual temperature of 28.4 °C ranging from 18.8 °C to 37.4 °C, and a relative humidity between 18 and 38%. The average annual rainfall is about 251 mm (Fig. 1). Water bodies such as rivers and streams normally exist in the area. The county is mostly mountainous and hilly, with scattered population inhabited mainly close to rivers. The villages are small and relatively difficult to access with a low population who live in houses made of cement blocks and shelters (Fig. 2). Farming and livestock herding are the main economic activities in this County. Malaria transmission occurs in this area year-round with peaks after the two rainy seasons (April-June and October-December), and *Plasmodium vivax* accounts for the majority of malaria cases [16]. In this region, IRS activities began in 1953, and since then at least two campaigns per year has taken place [2].

### Study design

This community-based cross-sectional study was conducted between January and March 2016 in Bashagard County.

### Sample size calculation

Assuming expected knowledge regarding malaria and IRS to be 50% and the desired precision of 5%, the sample size determined to be 420 [18].

### Data collection

**Fig. 3:**
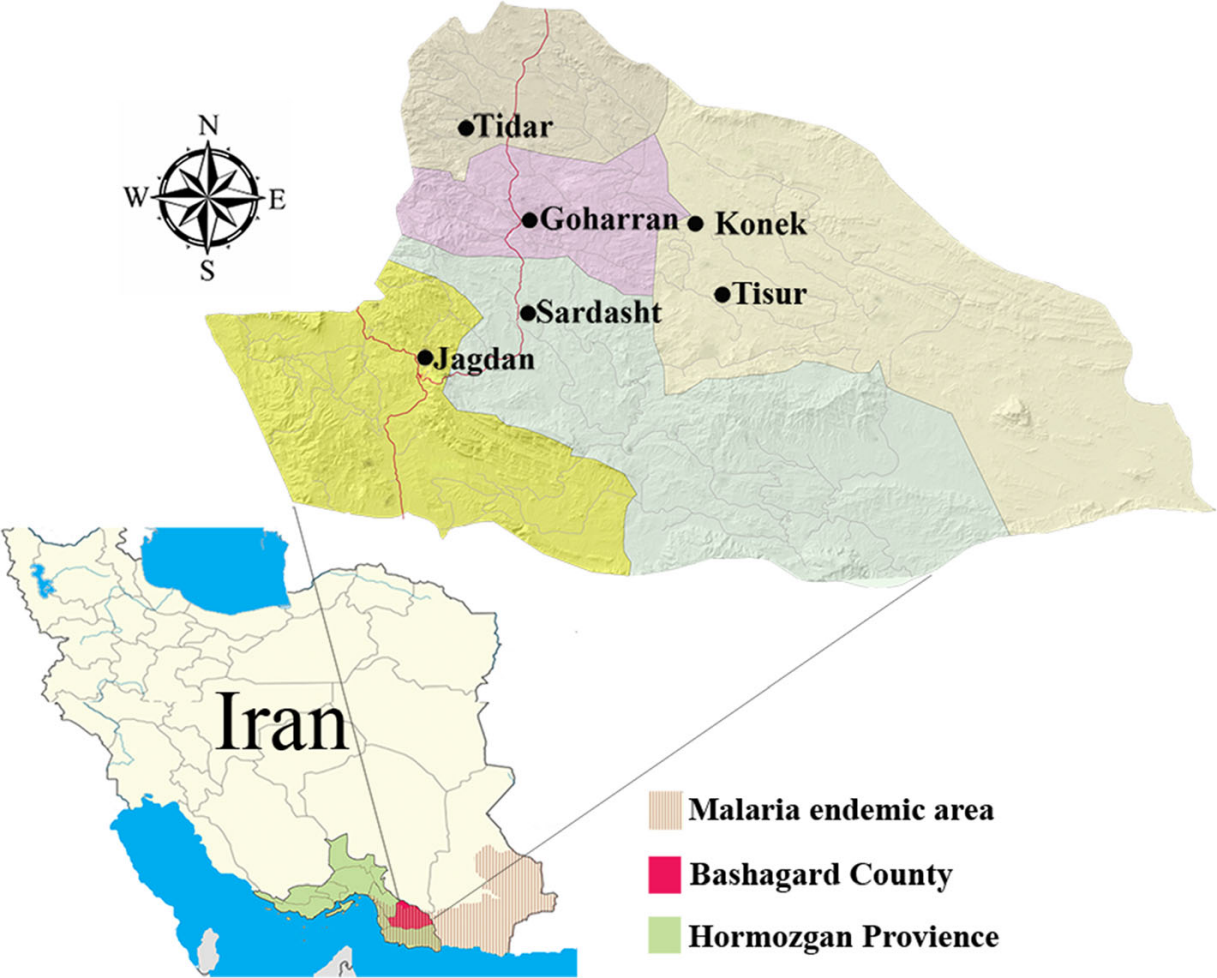
Map showing the provinces of Iran, highlighting the location of endemic malaria areas, and study villages in Bashagard County of Hormozgan Province, southeastern Iran

Inclusion criteria included being a permanent member of the community, being an adult (a woman or head of the household), and being resident in the sprayed houses. The exclusion criteria were being unable to communicate normally and non-cooperative households who refused to furnish necessary information. A two-stage randomized cluster sampling procedure was used to select the participants. In the first stage, six villages with similar epidemiological and topographical characteristics, where IRS has been ongoing, were randomly selected (Fig. 3). In the next stage, 70 households were selected from each village randomly. Since the fathers of families were mostly out of the house to work, mothers were interviewed using a pre-tested structured questionnaire. In case that mothers were absent, another adult member was interviewed instead. Questionnaires were filled via face-to-face interviews conducted by trained research assistants and supervised by the chief investigator.

The questions included respondents’ sociodemographic characteristics and knowledge and practices about malaria and IRS focusing on malaria symptoms and transmission, IRS coverage, the frequency of spraying, and positive and negative effects of the IRS programme.

As part of the data collection process, a checklist was completed through direct observations to investigate the use of IRS as well as housing conditions including water containers, indoor plumbing, construction materials, and status of windows.

### Statistical analysis

The data were coded and then analyzed using SPSS version 19. Descriptive statistics were used to determine averages, relative frequencies and percentages of the variables. Chi-square test was used to determine the association between the knowledge and practice regarding malaria and IRS and different variables. The results were considered significant at 5% levels of significance (*P* < 0.05).

## Results

### Socio-demographic characteristics

A total of 420 households participated in this study. The age of participants ranged from 17 to 75 years with an average of 29.8 years. The majority of the women had no formal education (55.8%), and 32.3% had completed primary school education. Most of the participants (96.5%) were unemployed and engaged in housework; others were self-employed, farmer/stockbreeder, and office workers. The average family size was 4.9 individuals and ranged from 1 to 11 people. Socio-demographic characteristics of the study population are illustrated in Table 1.

**Table 1:** Socio-demographic characteristics of the study population in Bashagard County, southeastern Iran

Characteristics	*n*	Percent
Age groups (years)		
15–24	66	15.6
25–34	126	29.7
35–44	98	23.1
45+	134	31.6
Education		
Illiterate	247	58.3
Primary	142	33.5
Secondary	10	2.4
High school	17	4.0
University	8	1.9
Family size		
1–2	55	13.0
3–4	138	32.5
5–6	133	31.4
7+	98	23.1
Occupation		
Housewife	409	96.5
Employed	4	1.0
Self-employed	9	2.1
Farmer/Stockbreeder	2	0.4

About 42% of households had a home constructed of cement blocks, and 8.3% of houses had screens over the window openings (Table 2). More than half of the participants were living in sheds which were made of palm leaves (56.8%), where domestic animals were mostly kept in the sheds (86.4%). About half of the population (50.7%) had access to piped water, 91.7% had electricity in their houses, and 60.4% had cooler (evaporative cooler or air conditioner) in their houses. More than half of the houses (62.5%) were built within 20 m of domestic animal shelters such as chickens, goats, and sheep (Table 2).

**Table 2:** Characteristics of residence houses in the study area in Bashagard County, southeastern Iran

Characteristics	Response: Yes		Response: No	
	*n*	Percent	*n*	Percent
Type of house				
Shed	241	56.8	183	43.2
Cement block house	179	42.2	245	57.8
Tent	4	1.0	420	99.0
Situation of house				
Window screens	35	8.3	389	91.7
Water supply	215	50.7	209	49.3
Water saving container	239	56.4	185	43.6
Electricity	389	91.7	35	8.3
Air conditioner	256	60.4	168	39.6
Animal shelter close to house	265	62.5	159	37.5

### Malaria knowledge and practices

The majority of the study population (85.5%) knew about malaria as a disease, and 85.4% of them knew that malaria is transmitted through a mosquito bite and level of knowledge was significantly associated with the level of education (*χ* ^2^ = 3.41, *df* = 4, *P* = 0.03) (Table 3). This study showed that some respondents had misunderstandings about malaria transmission causes considering the responses such as eating contaminated food, drinking dirty water, and inhaling polluted air they gave (Table 3).

**Table 3:** Knowledge and practices regarding malaria in the study population in Bashagard County, southeastern Iran

Parameters	*n*	Percent
Malaria transmission		
Mosquito bites	362	85.4
Drinking dirty water	19	4.5
Eating contaminated food	11	2.6
Inhaling polluted air	8	2.0
Do not know	32	7.5
Malaria symptoms		
Fever	344	81.1
Chill	58	13.7
Bone pain	19	4.5
Nausea	3	0.7
History of malaria infection in family members	226	53.3
Mosquito breeding places		
Stagnant water	318	75.0
Garbage	64	15.1
Do not know	37	8.7
Others	5	1.2
Malaria preventive measures		
Use of long-lasting insecticidal nets	205	48.8
Use of indoor residual spraying	144	34.3
Use of door/window screens	25	6.0
Chemoprophylaxis	21	5.0
Others	15	3.6
Noting	10	2.4
Interesting in participation in malaria control programmes	318	75.0

The most commonly mentioned symptoms of malaria were fever (81.1%), followed by a chill, joint/muscle pains, and nausea (Table 3). About half of the respondents (53.3%) mentioned having experienced cases of malaria infection in their family within the past 5 years (Table 3). The households who had a case of malaria infection in their family had a better knowledge of malaria symptoms compared to those with no history of malaria infection (*χ* ^2^ = 7.26, *df* = 4, *P* = 0.01).

Stagnated water considered by the majority of the respondents (75%) as breeding place of mosquitoes, although garbage also was mentioned as a breeding place (Table 3). Statistical analysis revealed a significant association between correct knowledge of mosquito breeding places and educational level of households (*χ* ^2^ = 4.28, *df* = 2, *P* = 0.001).

More than half (56.4%) of households reported they stored drinking water in containers inside the houses and 82% of them reported that they covered the water containers. The majority of respondents (83.3%) had LLINs, and approximately 69% reported that they covered the windows using mosquito screen.

Most of the participants reported LLINs, IRS, the screen on doors/windows, and chemoprophylaxis as preventive measures against malaria transmission (Table 3). The proportion of households with higher levels of knowledge on prevention and transmission of malaria that used preventive measures was significantly higher than in households with lower knowledge.

**Fig. 4:**
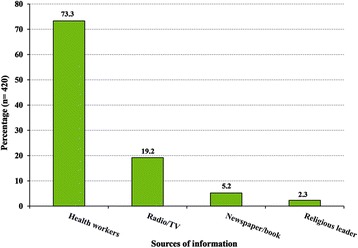
Sources of information about malaria and IRS in the study population in Bashagard County, southeastern Iran

Community health workers were reported to be the main source of households’ information about malaria and IRS. Other information sources were mass media including television, radio, newspapers, and books (Fig. 4).

### Knowledge and practice regarding IRS

This study’s results showed that most of the respondents (91.6%) had previously heard that insecticides were used for IRS. Out of those who had heard of IRS, 88.2% reported that IRS would be beneficial. A significant association was found between IRS acceptability and malaria knowledge of respondents (*χ* ^2^ = 6.42, *df* = 2, *P* = 0.002). In addition, most of the respondents (73.1%) mentioned that IRS should be conducted every six months and 1.9% said they did not know the frequency of IRS. Details of spraying frequency are shown in Table 4.

**Table 4:** Knowledge and practices regarding IRS in the study population in Bashagard County, southeastern Iran

Parameters	*n*	Percent
Ever heard of IRS		
Yes	385	91.6
No	35	8.4
Frequency of spraying		
Once in four months	26	6.2
Once in six months	307	73.1
Once a year	79	18.8
Do not know	8	1.9
Importance of IRS		
Prevention of mosquito nuisance	345	82.1
Prevention of other insects nuisance	42	10.0
Prevention of scorpion stings	24	5.7
Others	9	2.1
The exact parts of the house to be sprayed during IRS		
On the surfaces of inner walls and roof	198	47.1
On the surfaces of inner walls	135	32.1
On the surfaces of outer walls	31	7.4
On the inner surfaces of the roof	44	10.5
Do not know	12	2.9
Interesting in participation in IRS programmes as a volunteer	318	75.0

The results also showed that 26.7% of all the surveyed households had been sprayed in the previous summer and out of them, 65.6% reported that spraying was useful. The majority of the participants (96.6%) reported that several days before the spraying, they had received information about the IRS campaigns and community health workers were the main source of their information (67.8%).

**Fig. 5:**
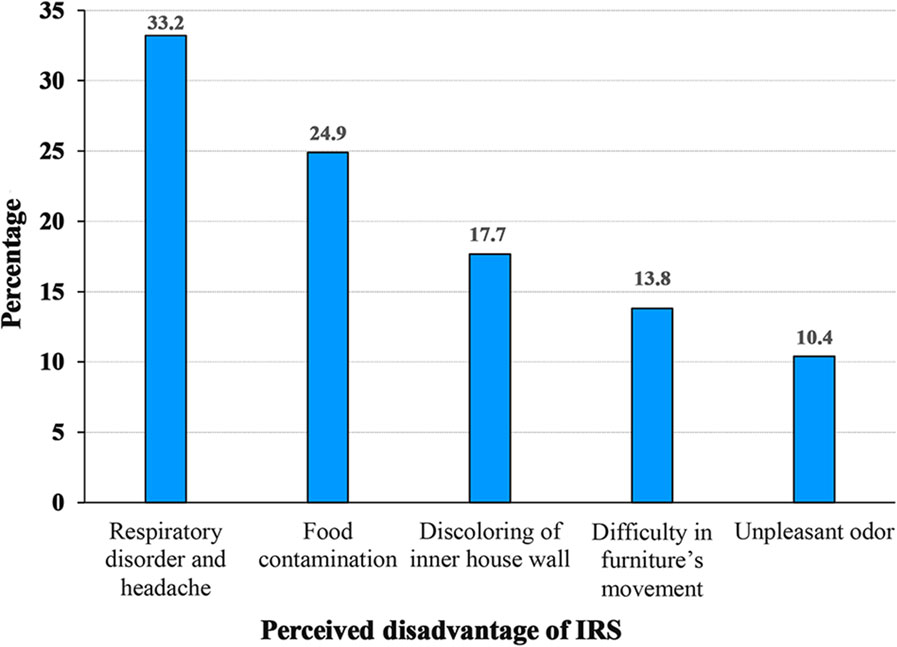
Dissatisfaction rate of the study population regarding IRS in Bashagard County, southeastern Iran

Protection against anopheline mosquito bites was reported to be the main reason for using IRS (82.1%). The other reasons were protection against other insects’ nuisance (10%), and scorpion stings (5.7%) and a few of the respondents (2.1%) reported that they did not know the importance of IRS application. The results revealed that there was a significant association between IRS acceptability and the level of education of households (*χ* ^2^ = 8.62, *df* = 6, *P* = 0.02). Some of the perceived negative health effects of IRS were an unpleasant odour, respiratory disorders, and headache. Difficulty in furniture’s movement, discolouring of inner house walls by insecticide, and contaminating the foods were other reported reasons for IRS refusal (Fig 5).

About 43% of study populations lived in simple houses made of cemented bricks with plastered cement walls. Most of the plastered cement walls (82.3%) were smooth with surfaces that lent themselves well to insecticide treatment. The results of the analysis indicated a significant relationship between the IRS acceptability and building materials (*P* < 0.001). Out of all households, 54.2% who were living in cemented brick buildings rejected IRS campaign. The exact parts of the houses to be sprayed with the insecticides are shown in Table 4. In this regard, most of the respondents (47.1%) mentioned that the surfaces of inner walls and roof should be sprayed.

According to the results, most of the respondents (75%) were interested in taking part in IRS campaign voluntarily (Table 3). A statically significant relationship was observed between the educational levels of households and their interest in participating in IRS programme as a volunteer (*χ* ^2^ = 4.12, *df* = 4, *P* = 0.032).

## Discussion

This study was conducted to provide baseline information on knowledge and practices regarding malaria and IRS which can be used in decision-making processes, the design of sustainable interventions with active community participation, and the implementation of educational programmes towards the prevention and control of malaria.

This study showed a high level of knowledge about malaria transmission and symptoms in the study population. Similar findings have also been reported from other malaria-endemic areas in the southeast of Iran [10, 14, 19]. Also, high awareness of people about malaria transmission and symptoms has been reported from other malaria-endemic countries including Malaysia, Saudi Arabia, Swaziland, Ethiopia, Ghana and Tanzania [17, 20–24].

Results of this study showed that Community Health Workers were the main source of household’s information about malaria and IRS. This finding is consistent with findings in other studies from different malaria endemic regions around the world which indicates that Community Health Workers are frequently in contact with people [21, 25, 26]. This is in contrast to findings from a recent study conducted in Saudi Arabia, India and Uganda that reported the social media as the primary source of malaria information [20, 27, 28]. Access to Community Health Workers and communication facilities had previously been reported to play an important role in prevention and control of malaria [29].

This study revealed a significant relationship between knowledge levels of the households about malaria symptoms and the history of malaria infection in the family. High awareness of the symptoms of malaria, which is a key to seeking early treatment, has been reported in populations in endemic malaria areas where people frequently suffer from malaria infection [25, 30, 31]. Understanding the treatment-seeking behaviours in populations will assist in identifying the possible barriers to surveillance and response activities that might exist such as reasons for delays in diagnosis and treatment. Moreover, understanding community behaviour about treatment-seeking will assist in the sustained community and health system efforts that will be required to prevent the resurgence of malaria following elimination [32].

In this study, the majority of the participants knew that mosquitoes transmit malaria and awareness about malaria transmission were positively associated with the age and seeking treatment. High levels of awareness about malaria transmission in the studied area can be explained by long-term exposure to malaria over the years and receiving information from health workers. Contrary to these findings, in other malaria-endemic countries such as Malaysia and Malawi only those with a higher level of education knew about the symptoms and vector of malaria [17, 33]. In this regard, results of some studies indicate that improving the community’s knowledge of malaria transmission can greatly contribute to prevention and success of control measures [17, 34].

Results of this study revealed that majority of the participants knew stagnated water as a breeding place of malaria vectors. This finding is consistent with findings in other studies in Iran which revealed high knowledge of people about mosquitoes breeding places [14, 35]. Similar results have been reported from other malaria-endemic countries such as Tanzania, and India [36, 37]. Awareness of mosquitoes breeding site could influence parameters which are involved in the vector control including the selection of residential areas and use of preventive methods aiming to decrease mosquito population density.

According to the results, more than half of households lived in poorly constructed houses and lacked window mosquito screens. Poor and inappropriate housing conditions have been proved to be associated with insufficient mosquito protection practices and a higher risk of malaria infection [25, 34]. Other studies have shown that in areas with low to moderate transmission, improving house design and using mosquito screens decreases mosquito densities and reduces malaria transmission [38, 39]. Furthermore, mosquito screens for houses are an appropriate, affordable, long-lasting, and acceptable protection method used in different communities [40].

This study indicated that despite having positive perceptions towards IRS, a large number of participants in the study population did not apply it. In this regard, 82.1% of the studied population mentioned IRS as an effective preventive measure against malaria vector, but only 26.7% of the houses had been sprayed as reported by the participants. This rate of IRS coverage is lower than 80% which is the targeted coverage by WHO [4]. Although this rate of IRS coverage is more than previously reported data from Iran [19], it is considerably lower than those reported form other malaria-endemic countries, such as Swaziland, Mozambique, Namibia, and Haiti [21, 41–43]. Therefore, the IRS coverage in the study area is much lower than the level required for effective control of the malaria vector.

According to our results, one of the explanations for such low IRS coverage can be a negative perception about it due to suspected IRS negative health effects, difficulty in furniture’s movement, and discolouring of inner house walls by insecticides. These results are consistent with the findings from other studies that reported insecticide smell, the mess left by the sprayers, the inconvenience of removing household items from houses before spraying as the causes of IRS refusal [44]. Moreover, other causes of IRS refusal have been reported to be poisoning of domestic animals, poisoning of children, and infertility of family [45]. Another reason for low IRS coverage in this study, as it has been reported from endemic malaria areas in Uganda, Yemen and South Sudan, can be the perceived low susceptibility and exposure to malaria infection as well as the perceived low severity of malaria infection in the community [44, 46, 47].

The effectiveness of IRS in malaria control is the main reason Iran adopted IRS as the main control strategy. However, it is of concern that IRS coverage was very low in the study population. This is especially so in light of the malaria elimination phase status which Iran carries. Therefore, efforts should be made for increasing the IRS coverage through promoting the attitude and practice of households regarding IRS for effective malaria control.

## Conclusion

There is a discrepancy between knowledge about symptoms, transmission route and control of malaria and use of IRS. Low socio-cultural and socio-economic status of the community are the main limitations to sustainable malaria elimination. These factors, along with community attitudes and practices, ultimately influence community participation in malaria elimination despite high knowledge. There is need to facilitate correct attitudes towards IRS use in Bashagard county if the disparity between knowledge and use should be bridged. Therefore, IRS campaigns accompanied with education for behaviour change should be considered as key elements for malaria control in the studied population. Moreover, continuous monitoring and evaluation of IRS and conducting more surveys on knowledge, attitude and practices are recommended to improve malaria control measures and to identify indicators for successful and sustainable malaria elimination programme.

## Abbreviations

IRS: Indoor residual spraying
LLINs: Long-lasting insecticidal nets
